# Serum neurofilament light chain predicts long term clinical outcomes in multiple sclerosis

**DOI:** 10.1038/s41598-020-67504-6

**Published:** 2020-06-25

**Authors:** Simon Thebault, Mohammad Abdoli, Seyed-Mohammad Fereshtehnejad, Daniel Tessier, Vincent Tabard-Cossa, Mark S. Freedman

**Affiliations:** 1 Ottawa Hospital Research Institute, University of Ottawa, The Ottawa Hospital – General Campus, 501 Smyth Road, Room 4118, Ottawa, ON K1H 8L6 Canada; 2 Department of Physics, University of Ottawa, Ottawa, Canada

**Keywords:** Multiple sclerosis, Prognostic markers

## Abstract

Serum neurofilament light chain (NfL) is emerging as an important biomarker in multiple sclerosis (MS). Our objective was to evaluate the prognostic value of serum NfL levels obtained close to the time of MS onset with long-term clinical outcomes. In this prospective cohort study, we identified patients with serum collected within 5 years of first MS symptom onset (baseline) with more than 15 years of routine clinical follow-up. Levels of serum NfL were quantified in patients and matched controls using digital immunoassay (SiMoA HD-1 Analyzer, Quanterix). Sixty-seven patients had a median follow-up of 18.9 years (range 15.0–27.0). The median serum NfL level in patient baseline samples was 10.1 pg/mL, 38.5% higher than median levels in 37 controls (7.26 pg/mL, *p* = 0.004). Baseline NfL level was most helpful as a sensitive predictive marker to rule out progression; patients with levels less 7.62 pg/mL were 4.3 times less likely to develop an EDSS score of ≥ 4 (*p* = 0.001) and 7.1 times less likely to develop progressive MS (*p* = 0.054). Patients with the highest NfL levels (3rd-tertile, > 13.2 pg/mL) progressed most rapidly with an EDSS annual rate of 0.16 (*p* = 0.004), remaining significant after adjustment for sex, age, and disease-modifying treatment (*p* = 0.022). This study demonstrates that baseline sNfL is associated with long term clinical disease progression. sNfL may be a sensitive marker of subsequent poor clinical outcomes.

## Background and rationale

Multiple Sclerosis (MS) is a heterogeneous neuroinflammatory and neurodegenerative condition for which short- and long-term prognostication is notoriously difficult. Some patients remain well for years with no treatment; others progress rapidly and are unresponsive to initial therapies^1^. In recent years, the repertoire of treatments on offer with higher efficacy has expanded, offset by some increased toxicity over previous baseline therapies^2^. In this context, accurate early prognostication is even more important as these highly effective therapies become more available^3^. If we can identify patients with more aggressive MS early on, we may be able to alter the trajectory of the disease, preventing or delaying the accrual of disability.

There is currently no accepted biomarker to indicate disease severity and inform choice of therapy. MRI provides an important yet non-quantitative snapshot^4^, and annual scans currently represent the standard of care in the assessment of disease activity and treatment response. However, this expensive and time consuming semi-quantitative imaging marker has limited sensitivity for predicting disease progression and prognosis^5^.

Neurofilament Light Chains (NfL) are neuronal specific intermediate proteins that are released from neurons and axons upon injury. In the CSF, NfL was discovered to be a biomarker of current and future disease activity^6^. Subsequent advances in assay technology enabled reliable quantification in the serum, a more practical biofluid, where levels are highly correlated with CSF levels, and also correlate with clinical and MRI disease activity^7^. A growing body of evidence indicates a role for NfL as a surrogate for disease severity, recent disease activity and treatment response^8–12^. Several studies have shown promising results as a short-term predictor of clinical outcomes and MRI changes up to 10 years later^13–17^. Here, we have investigated the prognostic value of serum NfL levels measured at an early-stage of MS for prediction of long-term clinical outcomes in a cohort followed up for more than 15 years, the longest follow-up to date.

## Objective

Are serum neurofilament light chain levels collected from MS patients within the first 5 years of symptom onset associated with worse clinical outcomes by 15 years, namely Expanded Disability Status Scale (EDSS) scores and and/or greater likelihood of reaching the progressive phase of the disease?

## Methods

### Subjects and controls

The Ottawa MS biobank comprises samples and data from patients followed by the MS clinic since 1994, who have consented their use for research purposes. Samples were collected at the time of patient’s diagnostic work-up and lumbar puncture and stored using a standard laboratory freezing method. On each visit to the Ottawa MS clinic (including baseline sampling visits) clinical data was collected including dates of first reported symptom onset, diagnosis and blood sampling and follow-ups, Expanded Disability Status Scale (EDSS) scores, clinical disease subtype (Clinically isolated syndrome or CIS, Relapsing Remitting MS or RRMS, Secondary Progressive MS or SPMS, Primary Progressive MS or PPMS), and any treatments given. Unfortunately, MRI data was not available consistently.

In this longitudinal cohort study, we screened our biobank for samples from patients who met the following inclusion criteria:

- Diagnosis of MS under 2010 McDonald criteria^18^
- More than 15 years clinical follow-up from disease onset
- Serum sampled (usually at the time of MS diagnosis) within the first 5 years of symptom onset

Controls were identified from available samples on patients who were of similar age and sex ratio at the time of sampling as the MS subjects. These controls had initially presented to the MS clinic for work-up of possible MS prior to being determined to have non-inflammatory ailments (migraine, fibromyalgia, chronic fatigue, and conversion disorder).

### Standard protocol approvals, registrations, and patient consents

Written informed consent had been obtained prior to inclusion in the Ottawa MS biobank. Approval was received from the Ottawa Hospital Regional Ethics Board for the measurement of serum NfL in this cohort of patients (OHSN-REB-20180518-02H). All methods were performed in accordance with these regulations.

### Sample collection, storage and serum neurofilament measurement

Serum samples were initially collected in red-topped serum tubes, and serum separated following coagulation by spinning at 2000×*g* for 10 min prior to aliquoting and storage in cryovials at − 80°. Prior to analysis, aliquots not previously thawed were selected and coded by a laboratory technician, blinding investigators to patient specific details; unblinding occurred only after analysis. All samples were thawed, processed and assayed as a single batch. NfL levels in each serum sample of patients and controls was quantified using commercially available NfL immunoassay kits (Quanterix, cat#103186) run on the fully automated ultrasensitive Simoa HD-1 Analyzer (Quanterix). Samples were run in duplicate in accordance with manufacturers’ instructions with appropriate standards and internal controls.

### Outcomes

The main objective of this study was to examine the association of baseline serum NfL and long-term outcomes of clinical disease progression (as defined below). However, we also compared baseline NfL with baseline clinical characteristics. Therefore, study outcomes were defined as follows:

1. Baseline clinical characteristics:
  - Timing of relapses around time of serum sampling
  - Baseline EDSS score (documented at the time of assessment)
  - Baseline disease subtype determined by retrospective chart review and application of the 2010 MacDonald Criteria (criteria were not available at the time of sampling)
2. Long-term clinical progression (main outcome)
  - Longitudinal EDSS score progression, defined as either of the following:
    1. A binary outcome of reaching an EDSS ≥ 4 and/or EDSS ≥ 6. Both are considered milestones in MS progression^19^, although natural history studies suggest that EDSS 4 is perhaps most relevant, after which MS progresses uniformly irrespective of preceding rate of disability accrual^20^
    2. Annualized rate of EDSS score progression (units per year, calculated as: first EDSS—last EDSS)/follow-up duration in years)
  - Developing progressive phenotype of the disease
    1. Neurologist diagnosis of primary progressive (PPMS) or secondary progressive disease (SPMS) determined by retrospective chart review and application of the 2010 MacDonald Criteria (criteria were not available at the time of sampling)

### Statistical analysis

This exploratory study had multiple aims and did not admit to a formal sample size calculation; we planned to analyze all available patient data that met our inclusion criteria. Nonetheless, with an assumed initial sample size of 100 patients per group, we determined that there was a 90% power to detect a standardized difference (Cohen’s d) of 0.66 between a dichotomous endpoint such as reaching EDSS ≥ 4, or developing progressive MS (Chi square).

All univariate and multivariate analyses, and statistical modeling were implemented using IBM SPSS Statistics software (version 23.0), Microsoft Excel 365 and Prism v.8.

1. *Data description* After assessing for normality of data using the D’Agostino and Pearson test numeric values, mean and standard deviation (SD) were used, whereas median and interquartile range (IQR) were applied to were used for non-normative data. Frequency percentages were used for description of categorical features.
2. *Exclusion of outliers* No outliers were excluded in the analysis
3. *Univariate comparisons* For univariate comparisons of normally distributed data, we used independent samples *t* test and one-way ANOVA where appropriate. For univariate comparisons of non-normally distributed data we used Mann–Whitney test, Kruskal–Wallis test or Chi square where appropriate.
4. *Multivariate data modeling* Generalized linear regression model was used to three steps to investigate whether the worse outcome of patients in the third tertile of serum NfL was confounded by other covariates. In all models, rate of EDSS progression (/year) was considered as the outcome variable and tertile of serum NfL at baseline was the main predictive variable. In the first step, a non-adjusted model was run, followed by an adjustment for age at the time of sampling. Lastly, a multivariate model was run including sampling age, sex, and disease modifying treatment as other potential confounders.
5. *Comparison of slope of progression* To compare the slope of EDSS progression over time between different tertiles of serum NfL at baseline, we used a repeated measures ANOVA test. Mauchly’s test of sphericity was applied to assess the homogeneity hypothesis of the error covariance matrix of the EDSS between groups. The Greenhouse–Geisser modification was used when the sphericity hypothesis was not assumed. We also adjusted the model for the confounding effect of the age.
6. *Receiver operating characteristics (ROC) curves* We assessed how accurately serum NfL at baseline predicts progression to EDSS ≥ 4, 6 or evolution to a progressive form of MS. For this purpose, we used receiver operating characteristics (ROC) curves, and calculated area under the curve (AUC) and its 95% confidence interval (CI). The best cut-off score was selected based on the value that maximised sensitivity and specificity at the same time using the Youden index. Finally, sensitivity, specificity, positive and negative likelihood ratios (LRs) were calculated for the best cut-off value of that biomarker level at baseline.
7. *Survival analysis* Kaplan–Meier method was applied to compare the average time to develop EDSS ≥ 4, 6 or conversion to progressive phenotypes between the tertiles of serum NfL at baseline. We used the Log rank test for between-group comparison and Cox regression model to calculate hazard ratios (HRs) for the outcomes of interest.

A two-tailed *p* value of < 0.05 was considered as the threshold for statistically significant association or difference.

## Results

### Participant flow and follow-up

**Figure 1:**
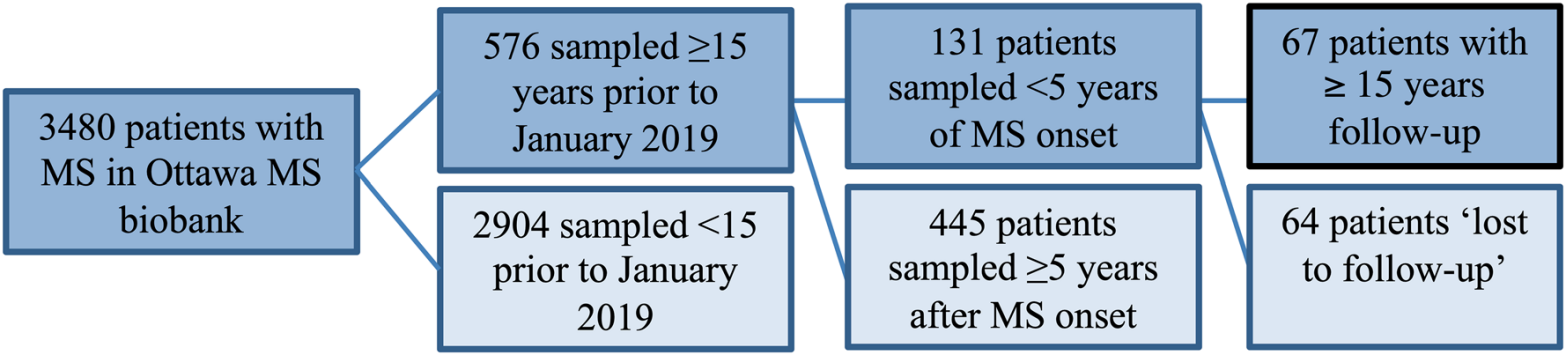
Patient flow.

As summarised in Fig. 1, at the time of sample selection and chart review in January 2019, there were 3,480 patients with a diagnosis of MS as per the 2010 McDonald diagnostic criteria and had a sample stored in the Ottawa MS biobank. Serum were collected since April 1994 and immediately stored in the same freezer at − 80°. Of the 3,480 patients, 576 had a clinical visit within 15 years but only 131 of these were seen initially within the first 5 years since symptom onset and were therefore eligible for study inclusion. 67/131 potentially eligible patients completed 15 years of follow-up in the Ottawa MS clinic (i.e. 64/131 or 49%, were ‘lost to follow-up’). All samples were thawed once on the day of NfL testing in January 2019. The baseline clinical and demographic characteristics of the 131 eligible patients were similar to the baseline characteristics of the 67 included patients:

- Of the 131 eligible patients, mean age at time of sampling was 39.5 years (IQR 13.75), 89/131 or 68% were female, median EDSS 1.5 (IQR1) (range), and 83/67 or 63.4% had positive oligoclonal bands.
- Of the 67 included patients, median age at time of sampling was 38.0 (IQR 14), 47/67 or 70% were female, median EDSS was 1.5 (IQR1), and 41/67 or 61.1% had positive oligoclonal bands.

Including baseline visits where serum was sampled, clinical status (including EDSS score, treatments, clinical subtype) was prospectively recorded on the 67 patients over a total of 560 separate clinic visits over 1,063.3 patient years between April 1994 and January 2019. The mean follow-up period was after serum sampling was 15.8 years (range 10.52–23.7); the mean follow-up from first MS symptom onset was 18.9 years (range 15.0–27.0 years). The median number of total visits per patient was 8 (range 6–12). The mean number of years between visits was 1.9 years (minimum 0.25 years, maximum 6.2 years). Patients who developed an EDSS score of ≥ 4 within the follow-up were seen on average 1.6 times as often as patients who remained EDSS < 4.

The 37 non-inflammatory controls were followed clinically for a mean of duration 2.2 years (range from 0 to 4 years) after baseline sampling prior to determination of final ‘non-inflammatory neurological’ diagnosis and discharge from the Ottawa MS clinic. All samples were collected on first clinic visits while these patients were being evaluated for MS during the same period as the MS patient serum samples, between April 1994 and January 2004. The median number of visits per patient was 3 (range 2–6). We performed a chart review at the point of inclusion into the study in January 2019 and determined that there had been no additional presentations concerning for organic and inflammatory neurological pathologies.

### Demographic and clinical data

Table 1 outlines relevant demographic and baseline clinical data on the 67 patients and 37 controls.

**Table 1:** Baseline demographic and clinical data of patients and controls.

	n	Sex F, M	Age at sampling (years)	Initial EDSS	OCB (n)	Disease course at sampling CIS/RR/PP	DMT started at sampling	Onset—last follow-up (years)	Diagnosis—last follow-up (years)	Sampling—last follow-up (years)
MS patients	67	47, 20	38 ± 14	1.5 ± 1	41	15/35/17	2	17.4 ± 3.64	16.06 (± 3.52)	15.8 ± 2.6
Final EDSS < 4	39	30, 9	35 ± 10	1.5(± 0.5)	22	3/37/5	0	17.7 (± 4.4)	16.2 (± 4.6)	15.7 (± 3.6)
Final EDSS ≥ 4	28	17, 11	43.5 ± 16	2.65 (± 1.50)	19	1/2/19	2	17.3 (± 3.42)	15.9 (± 3.5)	15.8 (± 2.64)
Controls	37	30, 7	38 ± 9	0	0	NA	0	NA	NA	NA

All ages, years and EDSS scores quoted as median ± IQR

*M* male, *F* female, *EDSS* Expanded Disease Disability Scale, *OCB* oligoclonal banding in CSF, *CIS* clinically isolated syndrome, *RR* relapsing remitting MS, *PP* primary progressive MS, *N/A* not applicable.

Baseline demographic details were similar between patients and controls, including ages and sex ratios. 18/67 patients had a clinical relapse within the 3 months before or after serum sampling, and 49/67 had not. At the time of baseline serum sampling, median EDSS score was 1.5 (range 0–5, IQR 1). Fifteen of 67 patients were diagnosed with clinically isolated syndrome (CIS), 35/67 with relapsing–remitting MS (RRMS), and 17/67 with primary progressive MS (PPMS). Two of 67 patients were already on injectable therapies (all interferon beta 1a). Of the 37 non-inflammatory controls, final diagnoses as follows: conversion/somatization (n = 14); migraine (n = 12); fibromyalgia (n = 6); anxiety (n = 5).

By the end of the follow-up (more than 15 years after baseline sampling), median EDSS was 2.5 (range 0–10, IQR 4.5). Twenty eight of 67 patients developed an EDSS score of ≥ 4 and 18/67 reached an EDSS of ≥ 6. The mean rate of EDSS progression was 0.1 EDSS points per year (range − 0.11 to 0.52). On each clinic visit, disease subtype was re-assessed based on clinical evolution of disease such that by 10 years, only 4/67 patients remained CIS, 39/67 had developed RRMS, 7/67 SPMS and 17/67 PPMS. By the end of the follow-up 40/67 had RRMS, 10/67 had developed secondary progressive disease (SPMS) and 17/67 had a diagnosis of PPMS, one of which had died from an unrelated malignancy. Twenty seven of 67 patients had received disease modifying treatments at some point during the follow-up period. At baseline serum sampling, 2/67 had received treatment (both with interferon beta 1a). 5 years after disease onset, 19/67 patients were on treatment (18 on injectable therapies interferon beta or glatiramer acetate, and one received mitoxantrone). At 10 years post disease onset, 21/67 patients were on treatment (15 on injectable therapy, 1 teriflunomide, 1 fingolimod, 1 mitoxantrone and one autologous haemopoietic stem cell transplant). By 15 years, 19/67 were actively receiving therapy (11 on injectable treatment, 2 teriflunomide, 1 dimethyl fumarate, 4 fingolimod, 1 natalizumab).

### Serum NfL levels compared to baseline clinical data

The distribution of NfL levels in both patients and controls was non-normally distributed (D’Agostino and Pearson normality test *p* < 0.0001). Therefore, averages are quoted as the median and interquartile range (± IQR) and comparisons were performed using non-parametric tests as described previously. In total, 5 patient baseline sera and 1 control had NfL levels > 3 × the interquartile range above the upper quartile. No data was excluded from subsequent analyses as these data represent plausible biological variation.

**Figure 2:**
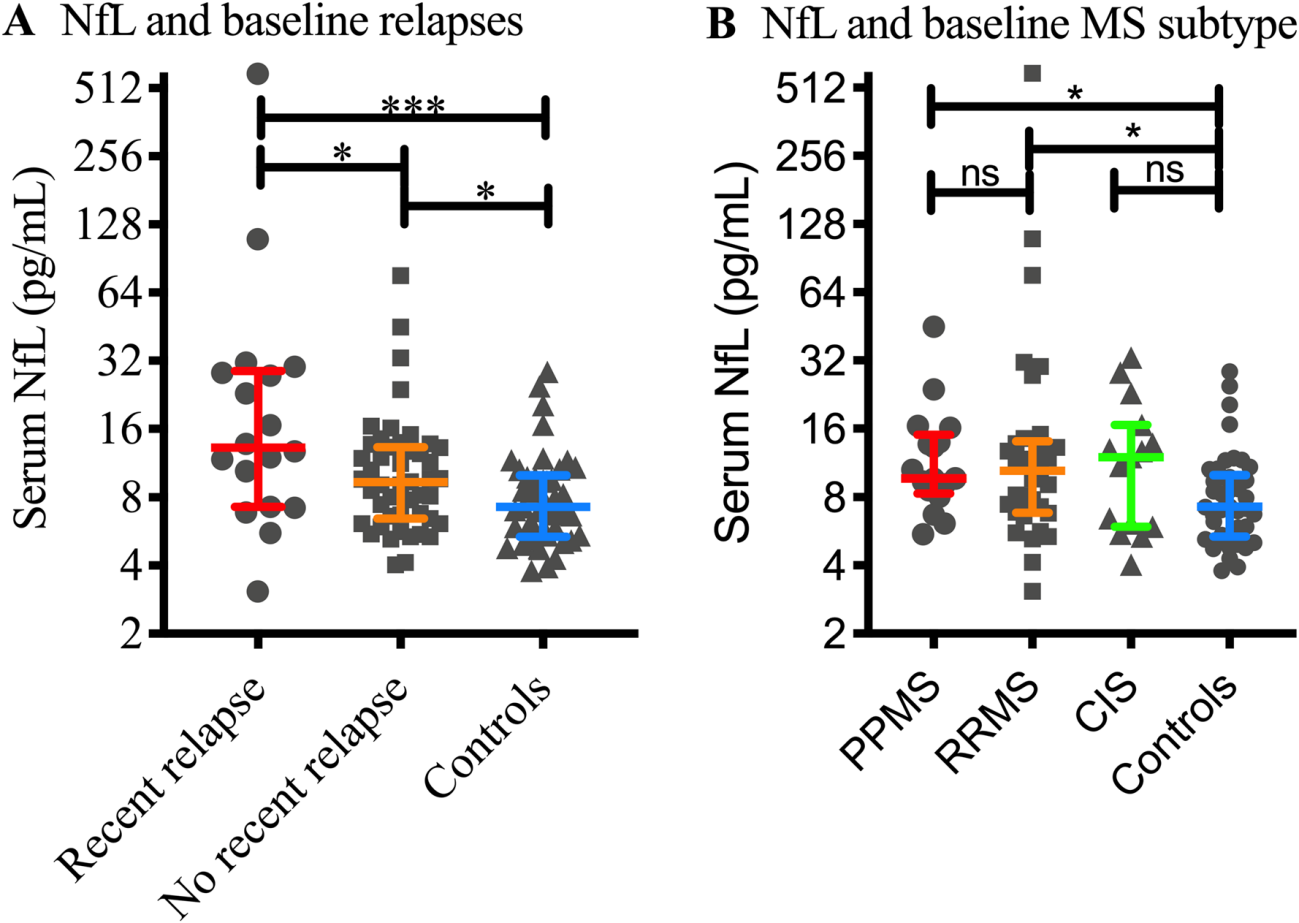
Raw serum NfL levels and baseline patient characteristics. Serum NfL levels were higher in patients who had had a documented relapse within 90 days of sampling compared to those who had not (*p* = 0.033, **A**) and controls (*p* = 0.0007). Patients who had not had a relapse within 90 days still had higher NfL levels than controls (*p* = 0.033). We did not find differences in NfL levels between different disease subtypes determined at baseline, although NfL levels were higher in both PPMS and RRMS patients compared to controls (*p* = 0.010 and 0.015 respectively). Y axis scale is Log(2) to most accurately represent the distribution of the data. Lines and bars depict median and interquartile range. Key: *PPMS* primary progressive MS, *SPMS* secondary progressive MS, *CIS* clinically isolated syndrome.

Baseline serum NfL levels were higher in MS patients as a whole (median NfL 10.06 ± IQR 7.61 pg/mL, n = 67) compared to controls (7.26 ± 4.62 pg/mL, Mann–Whitney *p* value = 0.004). This represents a 38.5% increase in patients relative to controls. As shown in Fig. 2A, median NfL levels in the 18 patients who had a clinical relapse within 90 days of serum sampling (13.24 ± 6.84 pg/mL) were 41% higher than the 49 patients who had not relapsed (9.36 ± 4.62 pg/mL, *p* = 0.033) and 82.4% increased relative to controls (*p* = 0.0007). We did not find an association between baseline NfL levels and absolute baseline EDSS score (Pearson r = 0.08, 95% confidence interval −  0.18 to 0.33, *p* = 0.53, data not shown). As shown in Fig. 2B, median NfL levels were similar between the 17 patients that were determined at baseline to have PPMS (n = 17, NfL = 9.68 ± 6.72 pg/mL), RRMS (n = 35, NfL = 10.44 ± 7.26 pg/mL) and CIS (n = 15, NfL 12.0 ± 9.8 pg/mL). Two patients received disease modifying therapies (both interferon beta 1a) at the time of sampling. Serum NfL levels were 8.1 and 9.6 pg/mL, both at the lower end of the MS patient measurements, however these were too few to enable subgroup analysis.

### Serum NfL levels and subsequent disease progression

**Figure 3:**
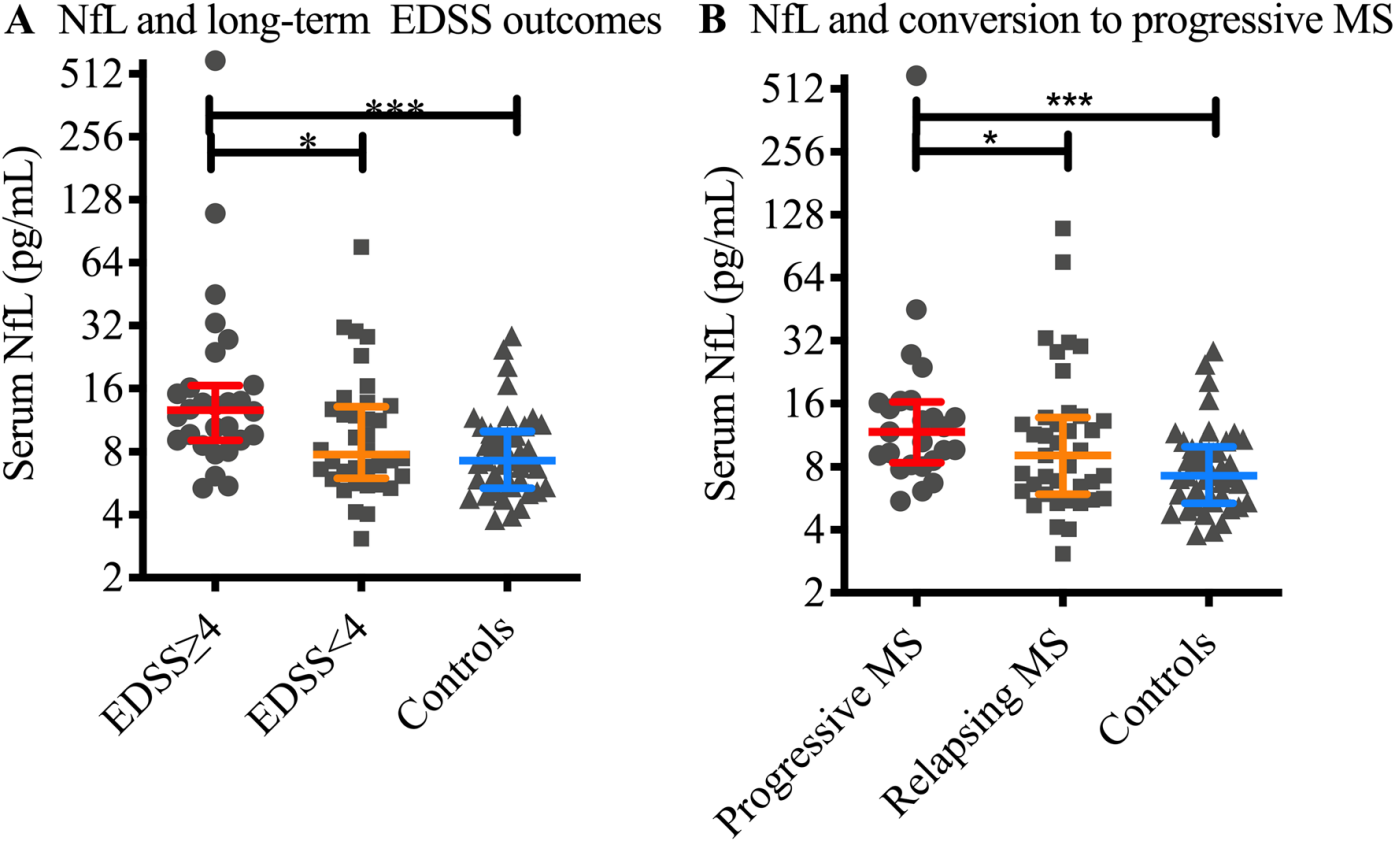
Raw serum NfL levels and subsequent disease progression. Serum NfL levels were higher in patients who reached EDSS ≥ 4 compared to those who had an EDSS < 4 (*p* = 0.0094, **A**) and controls (*p* = 0.0001), although was no significant difference comparing EDSS < 4 to controls. Similarly, baseline sNfL levels were higher in MS patients compared to controls, both in patients who developed progressive disease (*p* = 0.0005, **B**) and patients with relapsing disease (*p* = 0.041). Y axis scale is Log(2) to most accurately represent the distribution of the data. Lines and bars depict median and interquartile range.

As shown in Fig. 3A, after ≥ 15 years of clinical follow-up, median NfL levels in the 28 patients who had an EDSS score of ≥ 4 (12.6 ± 7.43 pg/mL) were 62.0% higher than 39 patients who remained EDSS < 4 (7.78 ± 7.11 pg/mL, *p* = 0.0094) and 73.6% higher than controls (n = 37, NfL = 7.26 ± 4.62 pg/mL, *p* = 0.0001). As shown in Fig. 3B, there was a trend towards higher median NfL levels in the 27 patients with progressive disease (NfL = 11.76 ± 7.88 pg/mL) compared to the 40 patients with longstanding relapsing disease (NfL = 9.08 ± 7.80 pg/mL), although this did not reach significance (*p* = 0.082). Relative to controls, levels in the progressive patents and relapsing patients were 62.0% (*p* = 0.0005) and 25.1% (*p* = 0.041), respectively.

### Receiver operating characteristics (ROC) curves

We compared the ability of baseline serum NfL levels to predict clinical outcomes of interest by the end of the follow-up using ROC curves (not shown) and found the following:

- For developing an EDSS ≥ 4, baseline serum NfL levels had an AUC of 0.734 (95% CI 0.63–0.84, *p* = 0.001).
- For developing an EDSS ≥ 6, AUC was 0.667 (95% CI 0.527–0,807, *p* = 0.038).
- For conversion to progressive MS (neurologist ascertainment of primary progressive or secondary progressive disease), AUC of 0.744 (95% CI 0.61–0.88, *p* = 0.054).

In a subsequent analysis using the Youden’s index method, a serum NfL value of 7.62 pg/mL was the best cut-off point to predict course of MS progression in long-term follow-up. Serum NfL value of 7.62 pg/mL showed a sensitivity of 88.9% (95% CI 70.8–97.6), and specificity of 47.4% (31.0–64.2), with a Positive Likelihood Ratio (PLR) of 1.69 (95% CI 1.21–2.35) and a Negative Likelihood Ratio(NLR) of 0.23 (95% CI 0.08–0.72 to predict EDSS ≥ 4; patients with levels less than 7.62 pg/mL were 4.3 times less likely to develop an EDSS of ≥ 4. Given that 28/67 patients reached this outcome (prevalence of 41.2%) the overall accuracy of this cutoff to correctly classify EDSS < 4 versus EDSS ≥ 4 was 66.2%. Meanwhile a higher cutoff of 13.2 pg/mL was associated with only 46.4% sensitivity (29.5–64.1) but 77.8% specificity (61.9–81.3), PPV 2.09 for reaching EDSS ≥ 4.

For conversion to progressive MS in long-term follow-up, baseline serum NfL value of 7.62 pg/mL demonstrated 93.3% (95% CI 68.0–99.8) sensitivity, 46.1% (30.1–62.8) specificity with an NLR of 0.14 (95% CI 0.02–0.99), PLR of 1.73 (95% CI 1.26–2.39) and accuracy of 63.1%. Patients with levels less than 7.62 pg/mL were 7.1 times less likely to develop progressive MS.

### Multivariate regression models

**Figure 4:**
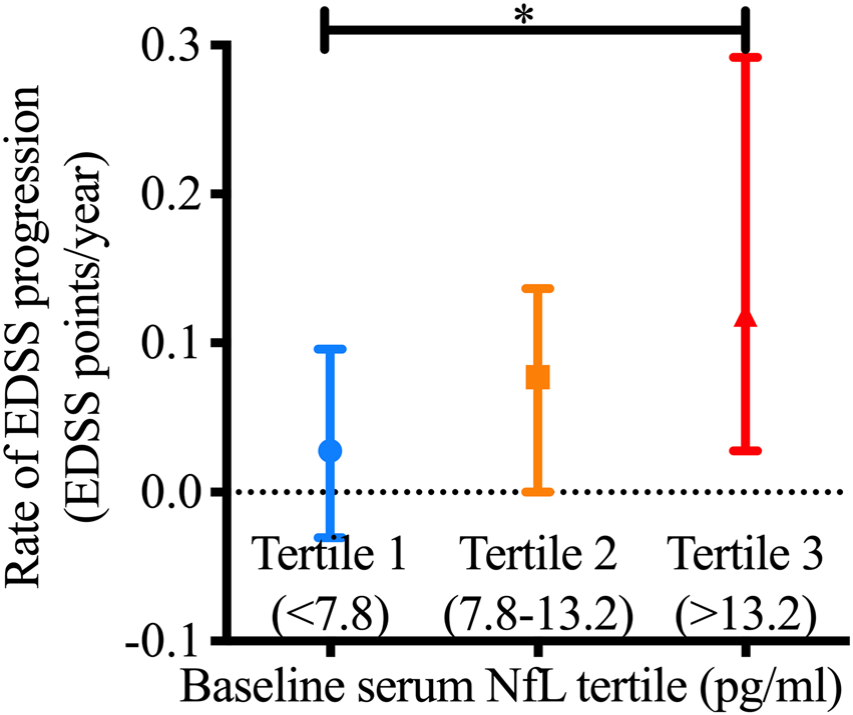
Baseline NfL tertile and rate of EDSS progression. Average rate of EDSS progression (median and Interquartile range) over time in subgroups of MS patients based on serum NfL tertiles at baseline (Kruskal–Wallis *p* = 0.020, *df* 2).

For evaluation of the dose-responsiveness, we divided MS patients into three equally-sized subgroups based on the tertiles of the serum NfL values at baseline. Patients with the highest values of serum NfL at baseline (3rd tertile, NfL level > 13.2 pg/mL), had the greatest median annual rate of EDSS progression, which was significantly larger than the other two tertiles (Fig. 4, 0.17 units/year, Kruskal–Wallis *p* = 0.020, *df* 2). This was also demonstrable in an unadjusted linear regression model; members of the 3rd tertile experienced on average a 0.125 further annual increase in the rate of EDSS progression compared to the 1st tertile (*p* = 0.004). As shown in Table 2, the association remained statistically significant after multivariate adjustment demonstrating that the association is independent of patients’ age, sex and disease-modifying treatment (model 3: B = 0.090, *p* = 0.022).

**Table 2:** Multivariate regression model of serum NfL tertile and rate of EDSS progression (points per year).

Serum NfL at baseline	Model-1 (unadjusted rate of EDSS progression)	Model-2 (age adjusted rate of EDSS progression)	Model-3 (age, sex, DMT and baseline EDSS adjusted rate of EDSS progression)
First tertile (< 7.8 pg/mL)	Reference (β = 0)	Reference (β = 0)	Reference (β = 0)
Second tertile (7.8–13.2 pg/mL)	β = 0.046, *p* = 0.287	β = 0.029, *p* = 0.511	β = 0.037, *p* = 0.346
Third tertile (> 13.2 pg/mL)	β = 0.125, *p* = 0.004	β = 0.117, *p* = 0.006	β = 0.090, *p* = 0.022

### Slope of progression

**Figure 5:**
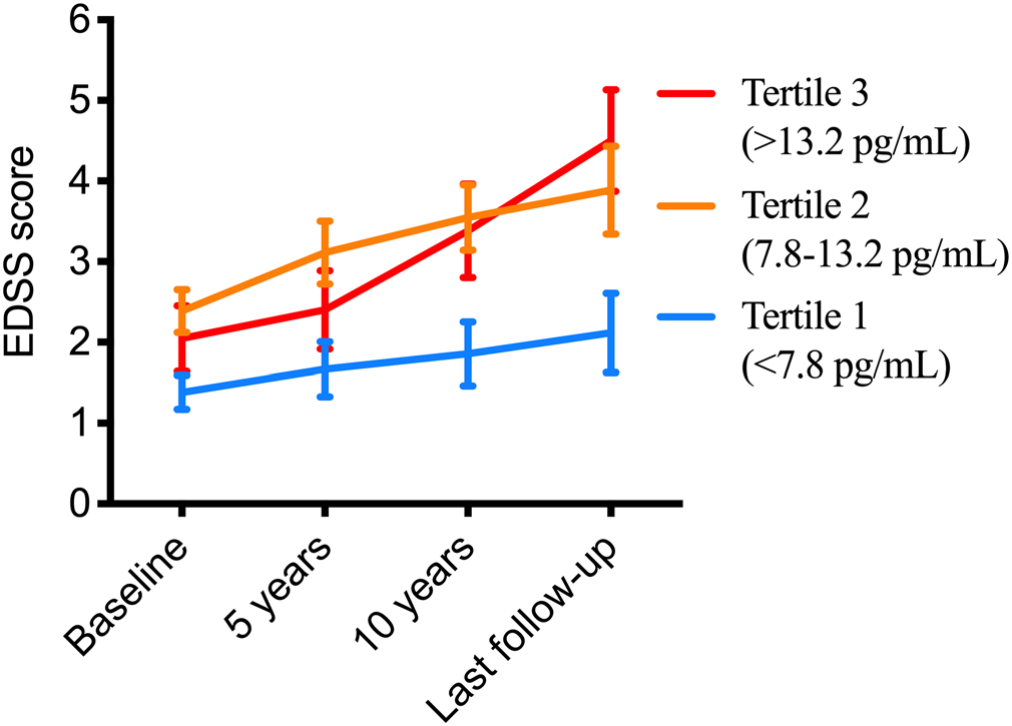
Baseline NfL tertile and EDSS trajectories. Trajectory of EDSS over long-term follow-up in subgroups of MS patients based on serum NfL tertiles at baseline (overall repeated measures ANOVA *p* = 0.031, *df* 2 *F* value 3.43).

Using repeated measures ANOVA, we compared the slope of progression in EDSS score over time between the tertiles of serum NfL. As illustrated in Fig. 5, MS patients with the highest (3rd tertile, range > 13.2 pg/mL) and lowest values of serum NfL at baseline (1st tertile, range < 7.8 pg/mL) progressed with the fastest and slowest pace, respectively. The time-tertile interaction was statistically significant (*p* = 0.0031), indicating that the curves were significantly non-parallel. This remained the case even after adjustment for the difference in patients’ age at baseline (Greenhouse–Geisser corrected *p* = 0.019).

### Survival analysis

**Figure 6:**
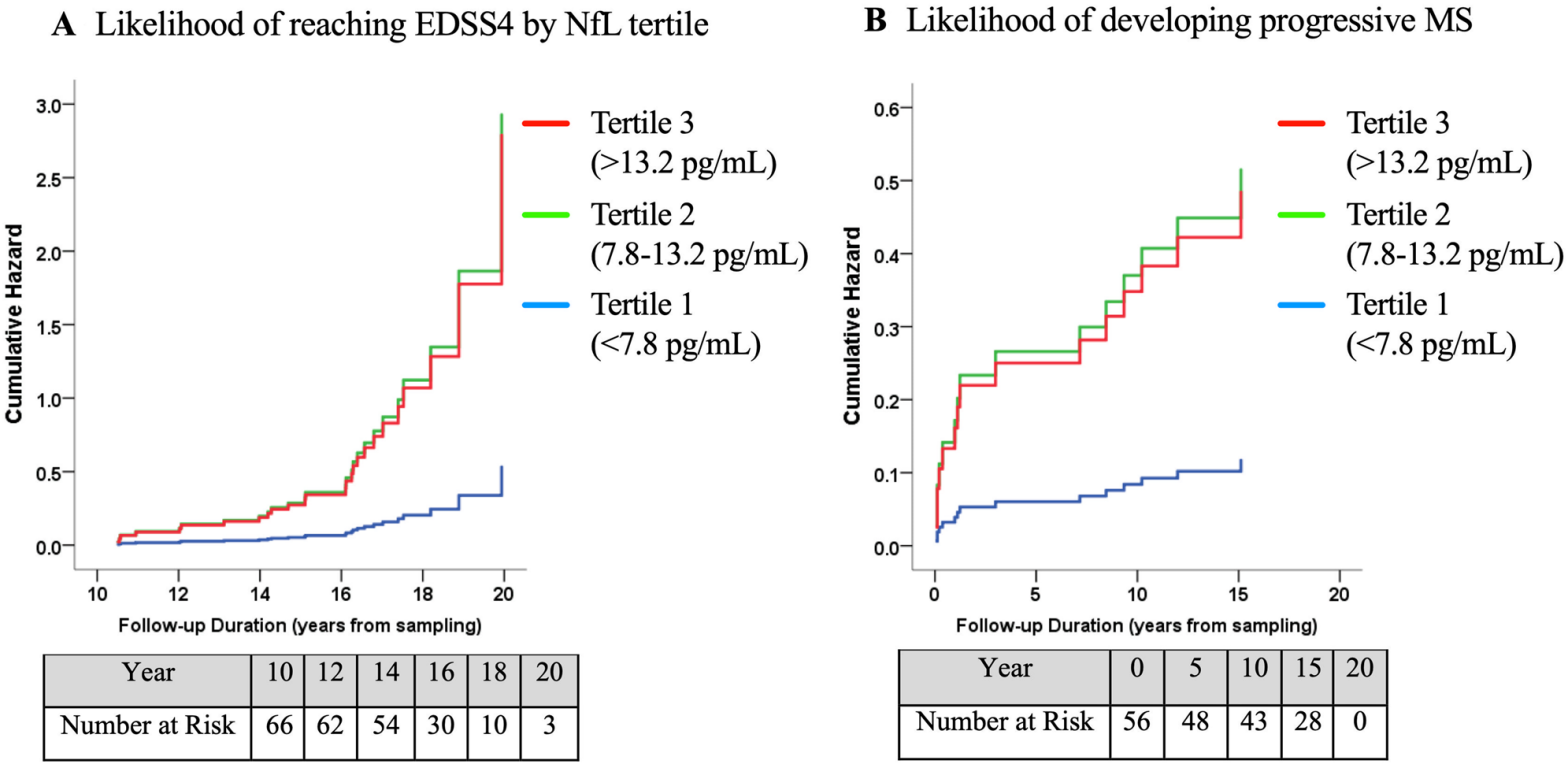
NfL tertile and likelihood of clinical progression. Kaplan–Meier survival curves to compare hazard of progression to EDSS ≥ 4 (**A**) (Cox regression overall *p* = 0.012 *df* 2 Chi-square 8.9) and conversion to SPMS (**B**) (Cox regression overall *p* = 0.0113 *df* 2 Chi-square 4.4) after long-term follow-up in subgroups of MS patients based on serum NfL tertiles at baseline.

We used Kaplan–Meier survival analysis and Cox regression model to compare time to reach clinical outcome between the subgroups. Compared to the 1st tertile with the lowest values of serum NfL at baseline, MS patients with higher serum NfL values in the 2nd and 3rd tertiles had a significantly higher hazard ratio (HR) of developing an EDSS ≥ 4 (2nd tertile: HR = 5.5 (95% CI 1.4–21.0), *p* = 0.012; 3rd tertile: HR = 5.2 (95% CI 1.5–18.6), *p* = 0.010), showing that they were on average > 5-times at higher risk of developing EDSS ≥ 4 over the follow-up (Fig. 6A). Sensitivity analysis demonstrated a similar trend for developing EDSS of ≥ 6 during follow-up. MS patients in the 2nd and 3rd tertiles of the serum NfL values at baseline had a larger HR for developing an EDSS ≥ 6 during follow up (2nd tertile: HR = 2.1 (95% CI 0.5–8.3), *p* = 0.307; 3rd tertile: HR = 3.6 (95% CI 1.0–13.4), *p* = 0.054, data not shown). For conversion to clinically diagnosed progressive MS, as shown in Fig. 6B, patients in the 2nd and 3rd tertiles had on average > 4-times higher risk to reach this endpoint compared to those with the lowest serum NfL values at baseline; however, this association failed to reach statistical significance with borderline *p* values (*p* = 0.065 and 0.082, respectively).

## Discussion

This is the first study to report an association between serum NfL levels obtained early in the course of MS and subsequent long-term rates of EDSS progression beyond 15 years. In this clinically heterogeneous group of patients, these associations remained significant after accounting for the confounding effects of age, sex, baseline EDSS score and exposure to disease. According to our study, the main utility of a one-off baseline NfL measurement is as a sensitive test that can rule-out patients who are least likely to progress. Patients with levels less 7.62 pg/mL were 4.3 times less likely to develop an EDSS score of ≥ 4 and 7.1 times less likely to be clinically noted to have developed progressive MS. Although having medium compared to high levels was not associated with worse outcomes, there was a statistically significant ordinal association between the annual rate of EDSS progression and baseline level of serum NfL.

The greatest criticism of serum NfL as a predictive biomarker in MS highlighted by this study is the significant overlap between baseline NfL levels in patients with MS and controls. While it is encouraging that there is a significant association between NfL levels and poorer outcomes at 15 years, and while we have shown the test is able to rule-out disease progression with reasonable accuracy, this is group level data. Based on this work, a one-off baseline sNfL level alone does not seem to be particularly helpful as a specific test to highlight individuals at greatest risk who would benefit the most from early and intensive treatment. We acknowledge that serum NfL levels represent one minor yet independent factor in determining overall MS risk of progression. Further work is imperative to optimise the positive predictive potential of NfL in individual placement. For instance, we anticipate that serial NfL timepoints and analysis of the change in NfL values rather than the absolute one-off value itself may be more informative.

Nevertheless, the quantity of NfL in the serum is rapidly emerging as a convenient and important biomarker in MS, with evidence for its role in monitoring disease activity and treatment response^7–9,16,21^. Although there is data to implicate its potential role early in MS in predicting short term outcomes^14–17,22,23^, the data for long-term outcomes less well established. Chitnis et al.^13^ found that serum NfL levels collected within 5 years of disease onset correlates with 10-year MRI markers including T2-weighted lesion volume and atrophy, but there was no association with 10 year EDSS. The patient cohort in this study was unusually benign, with only 11% of MS patients reaching an EDSS score of 3 or more by 10 years. Comparatively, our patients show a more aggressive course, with 28/67 patients (42%) reaching EDSS ≥ 3 by 10 years, and an even higher proportion by later timepoints. The more aggressive disease course and wider separation of long-term clinical outcomes in our cohort is reflected in our early serum NfL levels. This may be in part due to the inclusion of patients in our study starting from the early 1990’s at a time when lower efficacy disease modifying treatments (DMT) for MS were only starting to become available. Over the entire duration of the study, less than half of our cohort had received DMTs at any point despite many having developed advanced disease. If this study were repeated today, it is very likely that a much higher proportion of patients would receive one of the wide variety of MS treatments that are now on offer. This would likely confound interpretation of the prognostic biomarker quantification using baseline samples, as patients with aggressive disease are now often treated with more efficacious treatments, confounding the natural progression of disease and highlighting the value of historical biobanks such as ours.

We found that serum NfL cutoff of 7.62 pg/mL was the discriminator of future disease progression. A limitation of our method is that as both AUC and cutoff values for serum NfL were calculated using the same population, there is a chance for statistical inflation error in ROC analysis, and our finding needs to be validated on a separate cohort. Nonetheless, our cutoff of 7.62 pg/mL is very similar to level of < 8 pg/mL identified using the same commercial assay by Akgun et al.^24^, albeit in a different setting, as being associated with patients who sustainably met criteria for ‘No Evidence of disease activity’ (NEDA-3^25^) following alemtuzumab induction therapy. Natural history studies show that after EDSS 4, subsequent rate of disease progression progresses in a relatively uniform manner, regardless of prior relapses and rate of disability progression, further supporting the idea that in later disease, unlike earlier disease, progression occurs independently from inflammatory disease activity^26^. Early clinical factors known to be associated with a poorer prognosis include advanced age of onset, male sex, and rapid early disability accrual as well as progressive disease at onset^27^. In our analysis, the association of NfL and rate of EDSS progression was independent of these clinical confounders. In addition, the predictive value of serum NfL was independent of disease modifying medication exposure. The duration of the follow-up in this study spanned an era of significant drug discovery; while many of our patients were exposed to the first-discovered yet modestly effective injectable interferons and glatiramer acetate early in their disease, few had the opportunity to take contemporary higher efficacy therapies. Exposure to higher efficacy therapies may have distorted the natural history, especially if given early on.

The majority of patients and controls included in this study presented to the Ottawa MS clinic for initial evaluation for possible MS. Of the MS patients, only 2/67 were already on DMT, both interferon 1a. Our control population was made up of patients sampled in the same timeframe (1994–2004) who were only later determined to have ‘non-CNS inflammatory’ pathologies such as conversion disorder, rather than so-called healthy individuals. On the one hand, this can be seen as a strength of our study, as our non-inflammatory controls pragmatically resemble real-life clinical practice, where biomarkers are desperately required to discriminate MS from its mimics or other neurological conditions. On the other hand, as demonstrated in a recent metanalysis of CSF NfL levels^28^, even patients classified as having subjective neurological complaints may have higher NfL levels than truly healthy controls. The inclusion of an age and sex matched healthy control group might have helped eliminate this potential confounder.

There were other sources of potential error and bias which we could not account for in this pragmatic and retrospective study. Although the samples were collected near the time of diagnosis, disease onset was determined by asking patients to recall when they first developed symptoms, likely introducing inaccuracies. Given that many of these patients presented in the early 1990’s, we did not have digitized MRI in Ottawa as a comparative biomarker until May 2002, an important covariate. Furthermore, we demonstrated that relapses around the time of sampling resulted in increased NfL levels; serial NfL measurements would likely provide a more complete picture. Although the samples were stored carefully without freeze–thaw, and although the levels we measured in this cohort were similar to levels our group has measured previously in ‘fresh’ samples, the effect of long-term frozen storage on serum neurofilament levels is not known. Although we show that the baseline characteristics of the 131 eligible patients was very similar to that of the 67 patients who completed the 15 years follow-up, there was still a 49% loss to follow-up of eligible patients, introducing possible selection bias. Conceivably, patients who had more benign disease were more likely to become clinic non-attendees. Conversely patients with the more aggressive forms of MS resulting in mortality in the 15-year timeframe were also not included. We relied on clinical determination of EDSS scores and disease phenotype disease by multiple MS-specialised neurologists at several timepoints; although these tools are well validated in the hands of expert users, both have susceptible inter-rater differences as a source of error^29^. In this study we used a commercially available assay, whereas several other groups using the same SiMOA platform use a homebrew assay limiting comparability of absolute levels, highlighting the need for ongoing international validation work and consensus on serum NfL testing.

This study demonstrates group level data of the predictive value of early serum NfL levels over the lengthiest follow-up period to-date (an average of 16 years from MS diagnosis and sampling, and up to 24 years in some. One day, multimodal prognostic indices including clinical, MRI and serological data (such as NfL, perhaps most useful when measured serially) may assist in the identification of high-risk patients who may benefit the most from early aggressive therapies. Conversely, patients identified as having a very good prognosis may not require treatment at all, or will choose the safest and more modestly effective treatments. Better prognostication in early MS may enable physicians to be proactive rather than reactive, selecting appropriate treatment intensities prior to irreversible disability accrual, possibly altering the trajectory of disease and preventing progression.

## Data Availability

Consideration will be made by the authors regarding the sharing of anonymized data related to this study with qualified investigators.
